# Supplementary material for: Strength training among professional UCI road cyclists: Practices, challenges, and rationales
Source: PLoS One. 2025 Jul 10;20(7):e0328195. doi: 10.1371/journal.pone.0328195 (PMC12244580; doi:10.1371/journal.pone.0328195)
Supplement: S1 Fig — (DOCX) [file pone.0328195.s001.docx]

S1 Fig.

Survey: Strength Training and Cycling Performance

| Q.nr. | Question | Question type: Alternatives |
| --- | --- | --- |
| 1 | Year of birth | Type number |
| 2 | Sex | Single select: Male/Female |
| 3 | What is your current level of competition and team affiliation? | Single select: Junior, Club/elite team, Masters/>35, CTM, PRT, WTT, CTW, WTW |
| 4 | How many days do you reckon you competed/raced the previous season? | Type number |
| 5 | At what duration of effort would you consider yourself the strongest? | Multiple select: 1-30 sec, 30 sec-3 min, 3-8 min, 8-20 min, 20-60 min, 1h+ |
| 6 | Do you have a coach/coaches planning your training or giving you guidance? | Single select: Yes/No |
| 7 | Do you have a separate coach planning or giving you guidance on your strength training? | Single select: Yes/No |
| 8 | Does your coach incorporate strength training into your training plan? | Single select: Yes/No |
| 9 | How content are you with the guidance for your strength training? | Likert scale: 1-9 |
| 10 | How content are you with the guidance for your endurance training? | Likert scale: 1-9 |
| 11 | How much do you enjoy strength training? | Likert scale: 1-9 |
| 12 | How much do you enjoy cycling training? | Likert scale: 1-9 |
| 13 | Strength training frequency off-season | Single select: Sessions/week (0–7) |
| 14 | Strength training frequency pre-season | Single select: Sessions/week (0–7) |
| 15 | Strength training frequency race-season | Single select: Sessions/week (0–7) |
| 16 | Which muscle groups do you target in your strength training routine? | Multiple select: Upper body, Core, Lower body |
| 17 | What types of strength training do you perform? | Multiple select: Maximal, Hypertrophy, Explosive, Core/stability, BFR, CrossFit, Other |
| 18 | Rationale for performing strength training | Multiple select: Performance, Injury prevention, Fitness, Mass, Coach/team, Rehab, Bone health, Other |
| 19 | Challenges in maintaining a consistent strength training routine? | Multiple select: Fatigue, Time, Travel, Restarting, Knowledge, Coach’s knowledge, Motivation, Equipment, Other |
| 20 | What POSITIVE effects do you believe strength training could provide? | Multiple select: Performance, Sprinting, Injury prevention, Mass, Bone health, Health, None |
| 21 | What NEGATIVE effects do you believe strength training could provide? | Multiple select: Impaired performance, Fatigue, Injury risk, Weight, None |
| 22 | Confidence that strength training improves cycling performance? | Likert scale: 1-9 |
| 23 | Have you noticed any improvements from strength training? | Single select: Yes/No/Not sure |
| 24 | Additional comments or insights | Type text |
