# Supplementary material for: Strength training among professional UCI road cyclists: Practices, challenges, and rationales
Source: PLoS One. 2025 Jul 10;20(7):e0328195. doi: 10.1371/journal.pone.0328195 (PMC12244580; doi:10.1371/journal.pone.0328195)
Supplement: Attachment — Submitted filename: Professional road cycling stranght training gswinand rev1.docx [file pone.0328195.s002.docx]

Overall, this is a significant effort and interesting paper that deserves the chance to correct and resubmit.

The paper covers an important topic, i.e., how to incorporate strength training into a busy schedule, and how this might evolve over the season.

The paper collects a novel and important/interesting/likely unique dataset in that it surveys and interviews elite cyclists. The sample is substantial in size, covers both men and women, over time during the season, and has cyclists of several elite categories, e.g., world tour, conti pro, etc.

The paper gives a good but brief introduction to the relevant literature and lays out why this is important.

The paper is overall well written and to a very good standard of English.

The Authors state these are the questions they are looking to answer:

1) How do professional road cyclists integrate strength 83 training into their training routines? 2) What challenges related to their strength training do they 84 experience? 3) What is the rationale behind their decision-making related to strength training?

In spite of these positive elements, the paper needs revision to be of a peer reviewed publication quality. These criticisms are organised below in order of priority.

1) The biggest criticism of the paper is that the authors fail to make good use of what would appear to be good data and overall decent method. For example,
   a. the results of the scale and questionnaire –how is this incorporated? While table 2 gives quite a detailed list of items covered, the reader is then expecting to see how these items correlated or impacted the overall outcomes in terms of strength training; yet this is missing.
   b. Getting to table 3, we find that, perhaps unsurprisingly, cyclists did more strength training in the pre season than in the off season, and less still during the race season.
      i. Table 3 is really only comparing male vs female in terms of number of sessions/week; we find a very small difference between sexes in pre and during the season, but not in the offseason. While statistically significant, the order of the difference is 0.2x per week. Is this important? Does it inform coaches and amateur cyclists? Professional cyclists?
         1. Could this difference be driven by the fact that male cyclists likely race more days, race longer distances, races have more climbing, have more travel, and are perhaps more specialised in team role (e.g., sprinter, climber, etc?). The reader is left wondering.
   c. Table 4 again shows the types of strength training, but the statistics show only the statistical differences between male and females.
      i. Again the issue of core, vs upper and lower body and strength is confusing.
      ii. The categorisation of ‘explosive’ and ‘maximal’ – does this make sense? the overall categorisation has several categories for which the insignificance of the difference results are driven by the low numbers. It would make sense to group these and test the difference overall for different grouped categories.
   d. Table 5 shows the challenges to strength training but we are only testing the statistical difference between males and females;
      i. perhaps surprisingly, the only things that turn out significant is the travel time to races and camps.
      ii. The lack of interest seems to be driven by a small number in the females.
      iii. A number of interesting details of this table are not discussed – for example:
           1. Lack of coaches or facilities is not driving differences between males and females.
           2. Surprising muscle mass gain negative is not bigger for males.
           3. Almost 36% of females vs 27% of males say soreness is a negative, yet this difference is not statistically significant?
           4. I don’t understand the differences in the two results for the top part of the table on ‘Fatigue/soreness affecting the
           5. endurance training’ and ‘Soreness impairing endurance training’—how did the same question give such different results?
      iv. What table 5 really needs is analysis both across and down the columns; how do the different things stack up against each other?
          1. Then these results should be analysed including the other covariates the authors say they collected? What about UCI rank? What about age? Years prof? What about whether the rider is climber, sprinter or TT or Gregario/Domestique?
   e. Table 6 then the critique is more or less the same?
      i. We only see stat test of diff between sexes but so what?
      ii. Weak stat diff but no analysis down the table
      iii. We see for example ‘to improve sprinting performance’ but was the sample matched male/female in terms of how many sprinters vs climbers were male vs female?
      iv. Above all, we have the rationale for why they did it and then whether they think it worked – how did the matched sample compare across these? (did people achieve their goals in terms of perceived benefits?).
   f. There are added discussions which leave the reader wondering.

Starting at lines 259, they do some analysis of how content folks were with their coaching, etc, but Did coaching guidance have a benefit and help achieve success in terms of translating the perceived benefits into perceived success a la table 6?

Starting at 275, they state:

“275 Both female vs. male (Z= -2.051, p=0.04), those who trained strength during the racing season vs. those 276 who did not (Z= -4.001, p=0.001), and those who had a separate strength coach vs. those who did not (Z= 277 -3.148, p=0.002), was more confident that strength training would improve their cycling performance.” But this isn’t really telling us anything as no-doubt the riders who thought it ‘would work’ did more of it; but how did it turn out?

2) Some of the conclusions don’t seem to be adequately supported or contrast with the actual results, for example:
   a. ‘Coaching significantly influenced cyclists' strength training approaches. Most cyclists received 371 coaching, with many having a separate strength coach.’
      i. Does it really show this? Did we see the choices of types of strength training table 4 broken down by with/without coaching, or did they regress a multichotomous dependent variable on factors including a dummy variable for coach/no coach?
   b. “Male cyclists, who 323 compete more frequently, have shorter recovery and training times between races, that might contribute 324 to this difference.”
      i. Surprisingly, perhaps, the difference in table 5 is that a significantly higher % of women (c. 75%) say this is why they don’t do/face challenges to do strength training;

3) The selection of participants to the sample is self-selection. While the authors do not explicitly estimate treatment effects or draw strong conclusions about the population of UCI pro cyclists, there is a risk that the sample is significantly different based on self-selection. The authors should do some analysis of this, such as the % of male vs female respondents vs % contacted. Compare the mix or WT vs Conti tour; can they find the average age and UCI points of each type of rider and compare the population to the sample? If this cannot be done some effort or discussion and admission of limitations/potential bias is needed.
4) The choice of statistical method is not clear in some cases – why did they choose a chi-square? Why is this the correct test statistic? They appear to be only testing the differences between male/female proportions –why not choose a simpler test of the difference between two means or two proportions?
5) The paper could benefit from a more fine-tooth revision by a native English speaker. Some of the usage is a bit off and some of the meanings are unclear. I have not picked out too much, but for example,
   a. the first sentence contains an error, saying “Professional road cycling, a demanding endurance sport with varied races and multi-week stages” – the stages are not multi-week – stages are in a day; it is the race that is multi-week.
   b. 47 might be particularly important for cyclists on professional level who have shown to have lover levels of
6) The paper contains some small elements that could be clarified or made clearer in terms of the writing. For example,
   a. the authors seem to be drawing a distinction between ‘core’ and ‘strength’ training, but then mix things back up it seems later. 39 Core and stability strength training is popular….
   c. 172 “There were no significant differences between the male and female cyclists for which duration of effort the cyclists considered themselves to be the strongest, indicating that all cyclist characteristics was represented in both groups.”
