# Supplementary material for: Strength training among professional UCI road cyclists: Practices, challenges, and rationales
Source: PLoS One. 2025 Jul 10;20(7):e0328195. doi: 10.1371/journal.pone.0328195 (PMC12244580; doi:10.1371/journal.pone.0328195)
Supplement: Attachment — Submitted filename: Response to Editor and Reviewers.docx [file pone.0328195.s004.docx]

Response to Editor and Reviewers

Dear all:

Thank you very much for your comments and the opportunities to improve our manuscript. We have carefully reviewed each of your comments and tried to make changes according to your comments. Thank you very much for your constructive contributions; we believe this has improved the quality of the article and hope that the attached version will be suitable for publication.

Editor:

**Comment 1:** A review and revision should be performed by a native English speaker to clarify language, syntax, and grammar usage.

- **Response 1:** We acknowledge this suggestion and will ensure a thorough language revision by a native English speaker before final submission.

**Comment 2:** Clearly define, perhaps in a supplement different types of strength training and keep these distinct throughout the paper. Avoid mashing up the distinctions, as it gets confusing reading the paper; eg, strength and hypertrophy training generally result in similar adaptations, while specific “core” must be specifically defined and cannot be used synonymously with “stability” training, which should be defined. Moreover, how the “core” is defined muddies this.  Similarly, clearly define coach (sport vs S&C). Endurance coaches can and often do fill a strength coach role, but a “strength coach” or as distinguished by groups like the NSCA, the strength and conditioning coach, is a unique role.

- **Response 2:** In the questionnaire, "core and stability training" were combined into one category; therefore, separating them in the results is not feasible. However, we have revised the manuscript to ensure that distinctions between strength training types are clearer throughout the text, and we provide a definition of "core and stability training" in the introduction. Additionally, we have clarified the distinction between endurance coaches and strength & conditioning (S&C) coaches.

**Comment 3:** The authors should, as noted by reviewer 2, move the Informed consent/ethics section to near the beginning to make it clear that was performed first.

- **Response 3:** Revised as suggested.

**Comment 4:** The selection of participants to the sample is self-selection.  While the authors do not explicitly estimate treatment effects or draw strong conclusions about the population of UCI pro cyclists, there is a risk that the sample is significantly different based on self-selection.  The authors should do some analysis of this, such as the % of male vs female respondents vs % contacted.  Compare the mix or WT vs Conti tour; can they find the average age and UCI points of each type of rider and compare the population to the sample? If this cannot be done some effort or discussion and admission of limitations/potential bias is needed.

- **Response 4:** The ”Participants” section is revised based on the comments of editor and the reviewers for a more details and better clarification of the recruitment process. We have also revised the “Limitations” section so that it addresses these concerns. We do not have data to compare UCI points, but we found the number of riders in the whole population. We do not see it as necessary to include this in the study, but let us know if you think it should be included.

We also found the total number of cyclists for each category:

536 WT

404 PRT

1413 CTM (European teams)

240 WTW

667 CTW (European teams)

**Comment 5:** Secondarily, I note that there were just two women’s teams participating. Was there a team bias? IME across sports, the participation of strength training among endurance athletes in particular takes on a philosophical or dogmatic position, particularly among women. Do we know what the general practices/advice are among other teams? If not, it should be noted that not all teams adhere to similar training practices and this too may be a bias.

- **Response 5:** We would like to clarify that the study included respondents from two levels of women’s teams (Women’s World Teams and Women’s Continental Teams), not just two individual teams. We will revise the phrasing in the methods section to make this distinction clearer.

**Comment 6:** Both reviewers 1 and 2 ask for clarity in the methods, so please take head of this.

- **Response 6:** We have made our best efforts to revise the method based on the suggestions from both reviewers.

**Comment 7:** Please carefully consider the recommendations on results presented by reviewer 1, as well as the notes made by 1 & 2 on statistical aspects of the paper.

- **Response 7:** We have made changes to the results and the statistics as a result of the suggestions from the reviewers. This includes within-group analysis and the use of effect sizes and how these are interpreted.

Reviewer #1:

**Comment 1:** The biggest criticism of the paper is that the authors fail to make good use of what would appear to be good data and overall decent method. For example, the results of the scale and questionnaire –how is this incorporated? While table 2 gives quite a detailed list of items covered, the reader is then expecting to see how these items correlated or impacted the overall outcomes in terms of strength training; yet this is missing.

- **Response 1:** We appreciate the concern regarding the incorporation of the questionnaire and interview data into the results section. We would like to clarify that the key themes from Table 2 are thoroughly covered throughout the Results section, with findings supported by both quantitative data (e.g., statistical analyses of training frequency, challenges, and rationales) and qualitative insights from the most meaningful interviews.

For instance:

- Challenges related to strength training (e.g., fatigue, traveling, and race schedules) are detailed in Table 5 and further elaborated with direct interview quotes, such as a rider describing the difficulties of maintaining strength training while traveling for long race periods:

*"Suddenly, you can be away and staying in different hotels for 40-50 consecutive days, making it difficult to find a gym where you can execute a proper strength program."* (6PRT)

- Adjustments to strength training across seasons are presented in Table 3 and expanded upon in the interviews, where cyclists described their periodization approach:

*"We start with a conditioning phase with 10-12 repetitions, then gradually decrease after 4-5 weeks, down to 8 repetitions, and eventually down to 6 repetitions."* (3CTM)

- Rationale for strength training choices, including its perceived benefits for cycling performance, injury prevention, and bone health, is presented in Table 6 and supported by qualitative insights. For example, one cyclist highlighted the impact of strength training on bone density:

*"As a climber, it's undeniable that there's an impact on bone health, so I try to strengthen it as much as possible. And I have actually seen results."* (9WTW)

We believe that by integrating statistical findings with direct participant perspectives, the study presents a comprehensive and nuanced understanding of the data. However, we acknowledge the importance of making these connections more explicit and will consider minor adjustments in the Discussion section to further reinforce how the themes from Table 2 are represented in the results.

**Comment 2:** Getting to table 3, we find that, perhaps unsurprisingly, cyclists did more strength training in the pre season than in the off season, and less still during the race season.

i. Table 3 is really only comparing male vs female in terms of number of sessions/week; we find a very small difference between sexes in pre and during the season, but not in the offseason. While statistically significant, the order of the difference is 0.2x per week. Is this important? Does it inform coaches and amateur cyclists? Professional cyclists?
   1. Could this difference be driven by the fact that male cyclists likely race more days, race longer distances, races have more climbing, have more travel, and are perhaps more specialised in team role (e.g., sprinter, climber, etc?). The reader is left wondering.
- **Response 2**: We acknowledge that while the sex differences in strength training frequency during the pre-season and race season are statistically significant, their practical relevance requires further consideration. To better interpret the magnitude of these differences, we have replaced Z-scores with effect sizes in the results. Additionally, we address potential explanations for these differences, such as race days and traveling, in the discussion section rather than the results.

**Comment 3:** Table 4 again shows the types of strength training, but the statistics show only the statistical differences between male and females.

ii. Again the issue of core, vs upper and lower body and strength is confusing.
iii. The categorisation of ‘explosive’ and ‘maximal’ – does this make sense? the overall categorisation has several categories for which the insignificance of the difference results are driven by the low numbers. It would make sense to group these and test the difference overall for different grouped categories.
- **Response 3:** We have now included within-group statistics to asses the magnitude of the various variables. Regarding the classification of maximal and explosive strength training, we have maintained these as separate categories since they differ in execution and intent. Maximal strength training was defined by a low repetition range (<6 reps), whereas explosive strength training was characterized by lighter loads and an emphasis on maximal movement speed. Given these distinct training methodologies, we believe keeping them separate provides a more accurate reflection of training practices.

**Comment 4:** Table 5 shows the challenges to strength training but we are only testing the statistical difference between males and females;

iv. perhaps surprisingly, the only things that turn out significant is the travel time to races and camps.
v. The lack of interest seems to be driven by a small number in the females.
vi. A number of interesting details of this table are not discussed – for example:
    1. Lack of coaches or facilities is not driving differences between males and females.
    2. Surprising muscle mass gain negative is not bigger for males.
    3. Almost 36% of females vs 27% of males say soreness is a negative, yet this difference is not statistically significant?
- **Response 4:** We have now included within-group statistics to asses the magnitude of the various variables.

**Comment 5:** I don’t understand the differences in the two results for the top part of the table on ‘Fatigue/soreness affecting the endurance training’ and ‘Soreness impairing endurance training’—how did the same question give such different results?

- **Response 5:** Thank you for noticing this mistake! We had plotted the wrong number, but the mistake is now corrected.

**Comment 6:** What table 5 really needs is analysis both across and down the columns; how do the different things stack up against each other?

Then these results should be analysed including the other covariates the authors say they collected? What about UCI rank? What about age? Years prof? What about whether the rider is climber, sprinter or TT or Gregario/Domestique?

- **Response 6:** We have now included within-group analysis. We agree that this would be interesting to include data of UCI rank and other rider data, but this is unfortunately not included in this set of data and should be taken into account when interpreting the results.

**Comment 7:** Table 6 then the critique is more or less the same?

vii. We only see stat test of diff between sexes but so what?
viii. Weak stat diff but no analysis down the table
ix. We see for example ‘to improve sprinting performance’ but was the sample matched male/female in terms of how many sprinters vs climbers were male vs female?
x. Above all, we have the rationale for why they did it and then whether they think it worked – how did the matched sample compare across these? (did people achieve their goals in terms of perceived benefits?).
- **Response 7:** We have now included within-group analysis. Regarding the type of cyclists in each group. We did not ask them that type of cyclists they consider themselves, but we did ask them what duration of effort they would consider themselves the strongest. This included everything from short sprint durations to durations of over one hour. There were no differences between the groups for any of the durations as stated in the beginning of the results section. Regarding perceived benefits, in the second to last paragraph of the results we state that “Among the cyclists performing strength training, 65.4 % of male cyclists, and 79.6 % of female cyclists reported that they had noticed improvements in cycling performance from the strength training.”

**Comment 8:** There are added discussions which leave the reader wondering.

Starting at lines 259, they do some analysis of how content folks were with their coaching, etc, but did coaching guidance have a benefit and help achieve success in terms of translating the perceived benefits into perceived success a la table 6?

- **Response 8:** In the second-to-last paragraph of the results section, we report that 65.4% of male and 79.6% of female cyclists who performed strength training perceived improvements in their cycling performance. While this suggests a link between strength training and perceived success, the study did not specifically analyze whether coaching guidance directly influenced these perceived benefits.

**Comment 9:** Starting at 275, they state:

“275 Both female vs. male (Z= -2.051, p=0.04), those who trained strength during the racing season vs. those 276 who did not (Z= -4.001, p=0.001), and those who had a separate strength coach vs. those who did not (Z= 277 -3.148, p=0.002), was more confident that strength training would improve their cycling performance.” But this isn’t really telling us anything as no-doubt the riders who thought it ‘would work’ did more of it; but how did it turn out?

- **Response 9:** We acknowledge that confidence in strength training may influence engagement rather than directly indicating effectiveness. However, 65.4% of male and 79.6% of female cyclists who engaged in strength training reported perceived performance improvements. While this suggests a link between training and benefits, we recognize the limitation of self-reported data. Future research should assess objective performance measures to confirm these effects.

**Comment 10:** Some of the conclusions don’t seem to be adequately supported or contrast with the actual results, for example:

b. ‘Coaching significantly influenced cyclists' strength training approaches. Most cyclists received 371 coaching, with many having a separate strength coach.’
   i. Does it really show this? Did we see the choices of types of strength training table 4 broken down by with/without coaching, or did they regress a multichotomous dependent variable on factors including a dummy variable for coach/no coach?
- **Response 10:** We acknowledge that our original phrasing may have overstated the statistical evidence supporting the influence of coaching on strength training approaches. The questionnaire provided a quantitative overview of coaching prevalence and satisfaction, but it did not directly assess whether coaching impacted specific strength training choices (e.g., exercise selection in Table 4). However, the interviews offered additional context, as nine out of ten interviewees discussed the role of coaching in shaping their strength training practices. To clarify this, we have revised the discussion to put greater emphasis on the qualitative insights while ensuring that conclusions align with the presented data.

**Comment 11:** “Male cyclists, who 323 compete more frequently, have shorter recovery and training times between races, that might contribute 324 to this difference.”

ii. Surprisingly, perhaps, the difference in table 5 is that a significantly higher % of women (c. 75%) say this is why they don’t do/face challenges to do strength training;

- **Response 11:** We have revised the discussion to better reflect the data regarding the differences in strength training engagement between male and female cyclists.

**Comment 12:** The selection of participants to the sample is self-selection. While the authors do not explicitly estimate treatment effects or draw strong conclusions about the population of UCI pro cyclists, there is a risk that the sample is significantly different based on self-selection. The authors should do some analysis of this, such as the % of male vs female respondents vs % contacted. Compare the mix or WT vs Conti tour; can they find the average age and UCI points of each type of rider and compare the population to the sample? If this cannot be done some effort or discussion and admission of limitations/potential bias is needed.

- **Response 12:** The “Participants” section it now revised to provide more details around the recruitment process, as well as discussing limitations and bias in both the “Participants” and “Limitations” section.

**Comment 13:** The choice of statistical method is not clear in some cases – why did they choose a chi-square? Why is this the correct test statistic? They appear to be only testing the differences between male/female proportions –why not choose a simpler test of the difference between two means or two proportions?

- **Response 13:** We used the chi-square (χ²) test of independence to analyze between-group differences in categorical variables, such as sex differences in reported challenges to strength training. This test is appropriate for assessing whether the distribution of categorical responses differs between groups. We have now clarified this in the methods section to ensure transparency in our statistical approach. Initially, we focused on between-group differences using chi-square tests for categorical variables. However, to further assess differences in magnitude within groups, we have now conducted within-group analyses. Specifically, we used the McNemar test for categorical variables and the Wilcoxon signed-rank test for ordinal data. This additional analysis provides a more comprehensive understanding of how these variables differ both between and within groups. We have updated the methods and results sections accordingly.

**Comment 14:** The paper could benefit from a more fine-tooth revision by a native English speaker. Some of the usage is a bit off and some of the meanings are unclear. I have not picked out too much, but for example,

a. the first sentence contains an error, saying “Professional road cycling, a demanding endurance sport with varied races and multi-week stages” – the stages are not multi-week – stages are in a day; it is the race that is multi-week.
b. 47 might be particularly important for cyclists on professional level who have shown to have lover levels of
- **Response 14:** Thank you for noticing! Bot errors are now corrected. And the paper has now underwent a proofreading by an English speaker.

**Comment 15:** The paper contains some small elements that could be clarified or made clearer in terms of the writing. For example,

c. the authors seem to be drawing a distinction between ‘core’ and ‘strength’ training, but then mix things back up it seems later. 39 Core and stability strength training is popular….
- **Response 15:** We have now revised the part on core training to have a clearer definition.

**Comment 17:** 172 “There were no significant differences between the male and female cyclists for which duration of effort the cyclists considered themselves to be the strongest, indicating that all cyclist characteristics was represented in both groups.”

- **Response 17:** Revised for better clarity.

Reviewer #2:

ABSTRACT
**Comment 1:** Line 12; How many professional road cyclists were contacted? This should be clarified for the reader.

- **Response 1**: We have now added the number of cyclists contacted in the abstract and method.

**INTRODUCTION:**

**Comment 2:** Line 39; In the first paragraph the authors discuss the general benefits of strength training for cyclists / endurance athletes. At the beginning of the second paragraph, they refer to core and stability strength training. It would aid clarity for the reader if the authors defined the meaning of core and stability strength training and outlined how this is different from strength training discussed in the first paragraph. This is particularly important since the NSCA define core exercises as those involving recruitment of one or more large muscle areas involving two or more primary joints (multi-joint exercises), and that receive priority when one is selecting exercises because of their direct application to the sport. Does the author’s definition of core and stability strength training align with this or are they referring to a different type of training?

- **Response 2:** To improve clarity, we have added a definition in the introduction, specifying that in this study, it refers to training of all muscles between the shoulders and pelvis.

**Comment 3:** Line 40; The authors discuss findings of Sitko et al. Greater clarity is required here. What is meant by power across durations of 5 seconds to 20 minutes? How was power assessed?

- **Response 3:** Revised according to your comment to provide more details and better clarity.

**Comment 4**: Line 41; The authors should provide some examples to clarify their meaning of core and classical strength training exercises.

- **Response 4:** Revised according to your comment and added the exercises mentioned in the study.

**Comment 5:** Line 48; What is meant by bone markers? Are the authors referring to bone mineral density?

- **Response 5:** Revised based on your comment and added: “such as bone mineral density and bone mineral content”.

**Comment 6:** Line 73; The authors refer to previous work looking at the training practices of Tour de France finishers. Did the authors of this work clarify reasons for strength training not continuing six months prior to the event? This should be explicitly stated for the reader to provide greater rationale for the current work.

- **Response 6:** Added a sentence to clarify:” The study provided no further information on why the cyclists discontinued their strength training when approaching the race season.”

**METHODS:**

**Comment 7:** Line 87; How many cyclists were contacted? Were all cyclists on the FirstCycling.com database contacted and if not, how did the authors arrive at the specific cyclists to be contacted? How many cyclists responded and what was the response rate? Were all cyclists selected for interviews? How did the authors select cyclists for interviews, specifically was this a random or convenience sample?

- **Response 7:** 624 riders were contacted, and 147 responded to the questionnaire which gives a 23,5 % response rate. Due to limited time resources, we could not make effort to find other platforms to contact the riders that were not available via firstcycling.com database. The interviewees were selected from the riders that responded to the request on direct message, where we then followed with a second request for an interview. This continued until we reached the goal of 10 interviewees that covered all team categories. The method is revised for more information about these concerns.

**Comment 8:** Line 108; Insert ‘being’ after before.

- **Response 8:** Revised as suggested.

**Comment 9:** Line 117; The authors discuss collection of session frequency data collected via questionnaire and provide a scale of 0, <1, and 1 – 7 sessions per week. What is meant by less than 1 if 0 is provided on the scale? Is it assumed that each session lasts approximately 1-hour? Are there any questions about the duration of each strength training session?

- **Response 9:** The questionnaire focused solely on session frequency, not duration. The option "<1" refers to a strength training frequency of less than one session per week but still reflects regular engagement in strength training. For example, a cyclist performing strength training 2–3 times per month would select this option. In the statistical analysis, "<1" was treated as 0.5 sessions per week to provide a meaningful representation of their training frequency.

**Comment 10:** Line 142; Were all interviews conducted by the same person? How long did each interview last for each participant?

- **Response 10**: Added further details to the “Interview” section to assess your comment.

**Comment 11:** Line 161; The authors should move the section on Informed Consent and Ethical Practices to the end of the Participants section to clarify to the reader that informed consent and ethical approval was achieved.

- **Response 11:** Revised as suggested.

**RESULTS:**

**Comment 12:** Line 184; The authors state that a large percentage of cyclists ceased to perform strength training from pre-season to race season. This is repetitive (stated in line 180 also) and suggests that a large percentage ceased to perform strength training during race season. The authors should include the specific percentage in parentheses here also.

- **Response 12**: Removed sentence to avoid repetition, and added the percentage of cyclists that engaged in strength training during the various parts of the season.

**Comment 13:** Table 3; Z-scores are presented presumably looking at the magnitude of differences between male and female cyclists. Details of how z-scores were interpreted should be clarified in the Methods section.

- **Response 13:** Thank you for noticing this! We agree that the interpretation was not clear, and we have therefore decided to use effect size instead of z-scores. The interpretation is mentioned in the method section: “Interpretation followed the guidelines: small (r ≥ 0.1), moderate (r ≥ 0.3), and large (r ≥ 0.5).”

**Comment 14:** Line 200; The authors suggest that strength training practices “typically” comprised two weekly sessions. They should consider rewording to clarify that the athletes interviewed typically completed two sessions per week. The authors should clarify the specific season they are referring to since data is presented based on the training phase.

- **Response 14:** Revised according to comment.

**Comment 15:** Line 259; The authors report data suggesting the availability of a coach? Is this a strength and conditioning coach or a sports coach knowledgeable about strength training?

- **Response 15:** This referred to having a coach in general, which for most cyclists means having a cycling coach. This will in many cases be a coach that prescribes both endurance and cycling training. However, when we refer to “separate strength coach” we mean a coach dedicated to the strength training and not the cycling training.

**Comment 16:** Line 268; Is it meant that the “riders” that had a separate strength coach?

- **Response 16:** Typo corrected

**Comment 17:** Line 275; Check wording of this sentence.

- **Response 17:** Revised the sentence.

**Comment 18:** Line 282; Change “strength conditioning” to strength and conditioning.

- **Response 18:** Revised according to comment.

**DISCUSSION:**

**Comment 19:** Line 305; The authors should name the author for readability – i.e. Rønnestad and Mujika.

- **Response 19**: Revised as suggested.

**Comment 20:** Line 307; Change “in this study” to “in the current study”. This will aid clarity and create distinction from the previous study cited.

- **Response 20:** Revised as suggested.

**Comment 21:** Line 342; The authors should refer to the cyclists interviewed rather than many cyclists.

- **Response 21:** Revised as suggested.

**Comment 22:** Line 363; Check wording at the end of this sentence.

- **Response 22:** Revised as suggested.
