# Supplementary material for: Strength training among professional UCI road cyclists: Practices, challenges, and rationales
Source: PLoS One. 2025 Jul 10;20(7):e0328195. doi: 10.1371/journal.pone.0328195 (PMC12244580; doi:10.1371/journal.pone.0328195)
Supplement: Attachment — Submitted filename: Peer review r2 response.docx [file pone.0328195.s005.docx]

Peer review r2:

Editor comments:

Comment 1: Clarify your use of superscripts using either captions or foot notes to articulate data interpretation and when multiple superscripts are used.

Response 1: We have revised the captions for Tables 4–6 to clearly explain the use of superscript letters. These indicate within-group comparisons; values sharing a letter are not significantly different, while those with different letters are. When multiple superscripts appear, the variable overlaps statistically with multiple others.

Example: If one value is marked ᵃ, another is ᵇ, and a third is ᵃᵇ, the ᵃᵇ value is statistically similar to both the ᵃ and ᵇ values, but the ᵃ and ᵇ values differ significantly from each other.

Comment 2: Provide a bit more interpretive text regarding statistically significant data.

Response 2: We have revised the results section to include more interpretation of statistically significant results, focusing on what these differences mean practically for strength training among professional cyclists.

Comment 3: Avoid emotive terms in the text.

Response 3: We have reviewed the manuscript and removed or revised emotive language such as “whopping” to ensure a neutral and academic tone throughout.

Comment 4: Provide rationale for when road cyclists might desire hypertrophy; this last point is important, historically "bigger" or heavier is seen as negative to performance.

Response 4: We have expanded the discussion (L348-357) to provide additional rationale for why some road cyclists may pursue hypertrophy. While excessive muscle mass is generally seen as counterproductive in endurance sports, hypertrophy may be beneficial in certain cases — such as for sprinters or cyclists focused on power output. Additionally, some athletes may aim to maintain or prevent loss of muscle mass. We also acknowledge that due to the high training loads typical in professional cycling, substantial hypertrophy is often difficult to achieve, and gains tend to be modest.

Reviewer #1:

Comment 1: The presentation of the statistical results and differences with the superscripts is a good way, but in the caption or a FN spell out more that this is interpretable both down and across. (if i've understood correctly). What does it mean when there are more than one superscripts? Finally, some added interpretation of the results where things are stat significant.

Response 1: We have revised the captions for Tables 4–6 to clearly explain the use of superscript letters. These indicate within-group comparisons; values sharing a letter are not significantly different, while those with different letters are. When multiple superscripts appear, the variable overlaps statistically with multiple others.

Example: If one value is marked ᵃ, another is ᵇ, and a third is ᵃᵇ, the ᵃᵇ value is statistically similar to both the ᵃ and ᵇ values, but the ᵃ and ᵇ values differ significantly from each other.

We have revised the results section to include more interpretation of statistically significant results, focusing on what these differences mean practically for strength training among professional cyclists.

Comment 2: Give a good once over again, avoid emotive terms such as 'whopping'.

Response 3: We have reviewed the manuscript and removed or revised emotive language such as “whopping” to ensure a neutral and academic tone throughout.

Comment 3: for just road cyclists -why is hypertrophy ever 'good' or the goal?

Response 3: Response 4: We have expanded the discussion (L348-357) to provide additional rationale for why some road cyclists may pursue hypertrophy. While excessive muscle mass is generally seen as counterproductive in endurance sports, hypertrophy may be beneficial in certain cases — such as for sprinters or cyclists focused on power output. Additionally, some athletes may aim to maintain or prevent loss of muscle mass. We also acknowledge that due to the high training loads typical in professional cycling, substantial hypertrophy is often difficult to achieve, and gains tend to be modest.

Reviewer #2: 
Comment 1: INTRODUCTION: Line 60; typographical error. Check spelling of hypertrophy.

Response 1: Corrected the spelling error in ‘hypertrophy’.

Comment 2: RESULTS: Line 316; check wording of strengthening coaches here. Since strengthening is a verb, the authors are urged to refer to the strength (or strength and conditioning) coach. They should check the manuscript to ensure that they are referring to the strength (or strength and conditioning) coach consistently throughout – e.g. see Line 444 also.

Response 2: We have reviewed the manuscript and replaced all instances of “strengthening coach” with the correct term “strength coach” for clarity and consistency.
